# Should robots display what they hear? Mishearing as a practical accomplishment

**DOI:** 10.3389/frobt.2025.1597276

**Published:** 2025-09-26

**Authors:** Damien Rudaz, Christian Licoppe

**Affiliations:** 1 Department of Nordic Studies and Linguistics, University of Copenhagen, Copenhagen, Denmark; 2 Department of Economics and Social Sciences, Telecom Paris and Institut Polytechnique de Paris, Palaiseau, France

**Keywords:** automatic speech recognition, errors and mistakes, transparency, action ascription, conversation analysis, ethnomethodology, repair, mishearing

## Abstract

As a contribution to research on transparency and failures in human–robot interaction (HRI), our study investigates whether the informational ecology configured by publicly displaying a robot’s automatic speech recognition (ASR) results is consequential in how miscommunications emerge and are dealt with. After a preliminary quantitative analysis of our participants’ gaze behavior during an experiment where they interacted with a conversational robot, we rely on a micro-analytic approach to detail how the interpretation of this robot’s conduct as inadequate was configured by what it displayed as having “heard” on its tablet. We investigate cases where an utterance or gesture by the robot was treated by participants as sequentially relevant only as long as they had not read the automatic speech recognition transcript but re-evaluated it as troublesome once they had read it. In doing so, we contribute to HRI by showing that systematically displaying an ASR transcript can play a crucial role in participants’ interpretation of a co-constructed action (such as shaking hands with a robot) as having “failed”. We demonstrate that “mistakes” and “errors” can be approached as practical accomplishments that emerge as such over the course of interaction rather than as social or technical phenomena pre-categorized by the researcher in reference to criteria exogenous to the activity being analyzed. In the end, while narrowing down on two video fragments, we find that this peculiar informational ecology did not merely impact how the robot was responded to. Instead, it modified the very definition of “mutual understanding” that was enacted and oriented to as relevant by the human participants in these fragments. Besides social robots, we caution that systematically providing such transcripts is a design decision not to be taken lightly; depending on the setting, it may have unintended consequences on interactions between humans and any form of conversational interface.

## 1 Introduction

What should a social robot display about what it grasps from the world? Should this robot function as a complete “black box”, or should it make public some of the information it uses to generate its actions? Ought it to display how many humans it currently sees, the confidence score attributed to the presence of each human, or even—each time a human speaks—what its automatic speech recognition system grasped from this speech? Significantly, there can be no “neutral” or “hands-off” response to this inquiry. Any form of perceptual data made available by a robot during an interaction (raw data from its sensors or processed data) is a choice of design with potential interactional consequences: what a robot makes publicly available is one way (among many others) to select, process, and represent information it uses in local interactions.

The previous design dilemma is far from recent. A focus on what technological artifacts should describe, discretize, and communicate about their internal processes can be traced back at least to Norman (1988), for whom the surface visibility of action opportunities and of the state of a system were central concerns. As a direct extension of this line of inquiry, the question of the adequate degree of “transparency” for robots has recently received renewed attention. Namely, an emerging strand of studies focuses on what robots should display about their internal functioning (Chen, 2022; Fischer et al., 2018; Kong et al., 2024; Lee et al., 2023; Rossi and Rossi, 2024; Wortham and Theodorou, 2017). However, this question takes on a particular form with (intended to be) social robots: the act of clarifying what information is used by a robot to trigger specific behaviors on its part, or providing a real-time description of what this robot is currently “doing” (Fischer et al., 2018), seems, at first glance, capable of profoundly reconfiguring how humans interpret this robot’s conduct—i.e., which social action (Tuncer et al., 2022) these humans take the robot to be currently performing and if this action is relevant, inadequate, or erroneous. Even more, for robots aimed at being conversational partners (Irfan et al., 2024; Uchida et al., 2024), different degrees of transparency appear likely to heavily modify the usual informational ecology in which human conversations are ordinarily held. In this regard, a blind spot persists in debates about social robots’ transparency: the consequences of information made publicly available by a robot *on the relevance ascribed to its overall conduct* by humans.

**FIGURE 1:**
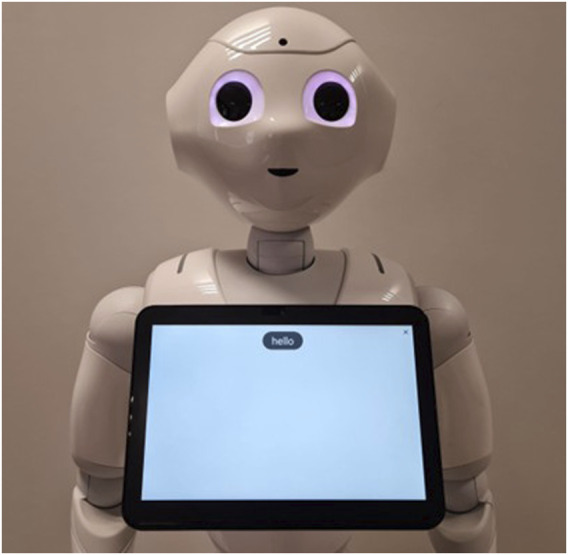
Default “speechbar” setting on a Pepper running its latest operating system (NAOqi 2.9). The robot displays “Hello” after a human pronounces this word and stops speaking for more than 200 ms.

Contributing to the literature on transparency and on failures in human–robot interaction (HRI), our study attempts to establish whether the informational ecology configured by displaying what a robot “hears” is consequential in the way miscommunications emerge and are dealt with compared with interactions that do not feature such information, including ordinary human conversation. To do so, we rely on a corpus of naturalistic and experimental interactions between humans and a version of the Pepper robot that displays the result of its “automatic speech recognition” (ASR) on the tablet placed on its torso. We preface our qualitative findings with an interesting pattern visible in the data obtained from our eye-tracking device: as the interaction unfolded, the attention of humans—materialized by their gaze—slowly focused more and more on the robot’s tablet (which exclusively displayed the transcript of what the robot heard, see Figure 1), while the robot’s head and gestures were gazed at less and less. Once this preliminary quantitative picture of participants’ gaze attention has been established, we use an ethnomethodological and conversation analytic approach to identify some local interactional phenomena aggregated in these eye-tracking data. Drawing on a detailed analysis of two video fragments, we show that the very emergence of “mistakes” or “errors” in the robot’s situated conduct was configured by what it displayed as having “heard”. Specifically, we explore how the “same” utterance or gesture of the robot (identical from an external and technical point of view) was treated by some participants as sequentially relevant only as long as they had not read the automatic speech recognition transcript, but was then re-evaluated as troublesome once they had read it. That is, we highlight how the automatic speech recognition transcript was used as a resource in framing the robot’s embodied or verbal conduct as inadequate.

The examined fragments constitute exemplars of situations where the robot did not properly “hear” what participants said, both from a technical point of view and as a situated phenomenon. Through their analysis, we emphasize that “mistakes” and “errors” can be approached as practical accomplishments that emerge as such over the course of human–robot interaction rather than as social or technical phenomena pre-categorized by the researcher in reference to criteria exogenous to the activity being analyzed. In doing so, we contribute to HRI by showing that systematically displaying an ASR transcript can play a crucial role in participants’ interpretation of a co-constructed action (such as shaking hands with the robot) as having “failed”. We observe that, as a specific form of transparency (Lee et al., 2023; Rossi and Rossi, 2024), an ASR transcript did not merely impact how the robot was responded to. Instead, it modified the very definition of “mutual understanding” that was enacted and oriented to as relevant by the human participants in our two fragments. We suggest that the automatic speech recognition transcript led these participants to enact a cognitivist definition of mutual understanding (as similar representations of the world) rather than an interactionist definition (as publicly mutually coordinated actions). Ultimately, besides social robots, we caution that systematically providing such transcripts is a design decision not to be taken lightly; depending on the setting, it may have unintended consequences on interactions between humans and any form of conversational interface.

## 2 Divergent informational ecologies

### 2.1 (Mis)hearing as a practical accomplishment

The following study relies on a long-standing ethnomethodological tradition that attends to the locally relevant features of a given setting as “accomplished” (Garfinkel, 1967; Pollner, 1974; Psathas, 1989); that is, as produced and maintained by co-present participants’ mutual conduct. This “study policy” (Garfinkel, 1967) investigates each property of a situation as emergent or, at least, treats it analytically “as if it were something that emerged from the activities of parties to that situation and that has no ‘existence’ independently of those activities” (Dennis, 2003). From this interactionist (Pelikan et al., 2022), emergentist (Rasmussen, 2019), or dialogical (Linell, 1995; 1998) perspective, no property of a participant’s conduct is, by itself and outside of any context, a “mistake” or an “error” (Peyrot, 1982; Zimmerman and Pollner, 2013). A conduct is constituted as troublesome only in and through the conduct of other participants to a setting, rather than as a “given”, before any interaction occurs (Meyer, 2019; Pollner, 1974). According to this methodological standpoint, interactions do not feature ontologically “erroneous” actions that are merely detected by co-interactants: “errors” are emergent categories (Mondada, 2017; 2018) displayed in participants’ orientation towards a local situation. It is within the immediate context of the interaction, and often through documented conversational methods (accounts, repairs, corrections, clarifications, etc.), that a “mistake” is constructed as such (Albert and Ruiter, 2018; Schegloff, 1992).

This interactionist stance implies a specific understanding of what it means to “(mis)hear” an interlocutor. As a matter of fact, human recipients do not display on their foreheads the exact words they hear (and potentially mishear) during other humans’ speaking turns. A somewhat obvious consequence of this state of affairs is that, when, from an internalist point of view, an interlocutor completely “mishears” another participant’s speaking turn, this is not an accountable phenomenon of miscommunication in itself: the only available resource for a co-present participant to detect and repair a potential “mishearing” is this interlocutor’s embodied or verbal *response* to the previous turn (Mondada, 2011). If, in a noisy place, a participant happens to perceive absolutely nothing of their interlocutor’s voice during a conversation, they may still fortuitously produce a second pair part of the type and form normatively expected (Kendrick et al., 2020) after the previous turn of this interlocutor (for example, by responding with a greeting to a greeting, or by laughing after a joke). In turn, this response may be treated, by co-present participants, as adequate to the action produced by the previous speaker. Should this occur, for all practical purposes, from the strictly emic perspective of participants involved in this ongoing conversation, these conversationalists’ conduct is coordinated, and nothing stands out as “repairable” on the surface of interaction (Albert and Ruiter, 2018; Jefferson, 2017). In other words, by themselves, the cognitive processes of a co-participant are not pragmatically consequential phenomena: when a conversationalist is treated by co-present participants as “having (im)properly heard someone” or as “having understood an instruction”, it is not this conversationalist’s mental activity (as a presumed “private process”— (Wittgenstein, 1953)) that is being indexed as constituting “(im)proper hearing” or “proper understanding” (Coulter, 1999; 2008). This conversationalist’s (in)adequate subsequent conduct *will be what “(mis)hearing” is* (Coulter, 2008; Ryle, 1949) rather than the mere evidence of a postulated underlying mental state.

### 2.2 Human–robot informational ecologies

A fundamental feature of ordinary human–human interactions is that what is “inside people’s heads” is not a publicly available resource for participants involved in a local situation (Deppermann, 2018; Garfinkel, 1963; Kristiansen and Rasmussen, 2021). As humans “do not carry MRI machines with them out in the world” (Kerrison, 2018), what happens in conversationalists’ brains is never oriented to by their interlocutors as a relevant property of the setting. In most face-to-face interactions, humans’ inner cognitive or physiological processes are rarely made systematically transparent to other interactants by being displayed on a screen: namely, in ordinary conversations, humans do not display real-time transcriptions of what they are “hearing”, “perceiving”, “picturing”, etc. In these elementary conditions in which social interaction takes place, one parameter remains constant: whether for the involved actor or the researcher studying video data, participants for whom this would be a practical concern must always rely on “inferential procedures” (Deppermann, 2012) to establish relationships between “discourse and cognition” (Deppermann, 2012)—between what one’s interlocutor says and what they may “think”, “see”, “feel”, “perceive”, etc^1^. Moreover, several works have noted that, while they are immersed in the practical urgency of their daily interactions, conversationalists are ordinarily seldom interested in detailing the exact phonemes, words, and gestures that they take their interlocutor to have “perceived”, “processed”, mentally “represented”, etc., (Albert and Ruiter, 2018; Dingemanse et al., 2023; Mondada, 2011). Participants in a conversation are generally not concerned “with attaining absolute terminological precision as in certain scientific genres” (Linell and Lindström, 2016).

Yet, this specific informational ecology of human–human interactions appears not to translate entirely to a substantial part of human–agent interactions: those where the agent provides a written trace of what it “heard” the human say. For example, for the commercial humanoid robot Pepper, the nominal behavior is to display on its tablet (attached to its torso) a transcript of what human interlocutors are saying, as “heard” by the robot. The top of this robot’s belly screen features a “speechbar”^2^ where the result of the automatic speech recognition will be written once the robot hears no more speech for more than 200 ms (see Figure 1).

Significantly, at first glance, publicly displaying the result of the “automatic speech recognition” appears to open a window on what is going on “inside the robot”, before this robot produces any verbal or embodied response (speech, sound effects, gestures, LEDs, etc.) to the previous turn of an interlocutor. For example, a robot that was just greeted with “hello” will display “hello” on the tablet before it starts its return greeting action. In this view, human participants have direct access to the exact receipt of the words they have pronounced before any return action from the robot can be achieved. When treated as such, i.e., as intended by its designers^3^, an automatic speech recognition transcript reconfigures the informational ecology of the interaction: it becomes possible for a participant to pinpoint if the upcoming action of the robot stems from a correct receipt of the words of the previous turn. Hence, this type of informational configuration currently appears to have no counterpart in interactions that do not comprise robots or conversational agents. There exists no so-called “human–human” face-to-face configuration involving a *scriptural resource* displaying precisely what words were *heard* by an interlocutor in real time^4^. These non-overlapping informational configurations (between interactions involving ASR transcripts and those that do not) belong to the wide set of parameters for which human–robot communication have been described as asymmetric (Frijns et al., 2023).

## 3 Related work

A common and long-documented challenge for roboticists and users alike is that robots frequently struggle to properly hear human speech when deployed in noisy environments outside the laboratory (Addlesee and Papaioannou, 2025; Foster et al., 2019). Several studies have explored these ASR failures in interactions with robots and vocal user interfaces (Förster et al., 2023; Gunson et al., 2022; Luger and Sellen, 2016) and how the resulting interactional trouble is handled by users (Fischer et al., 2019; Porcheron et al., 2018). However, to the best of our knowledge, no study has yet been undertaken about the specific impact of (erroneous) speech *transcripts* on human–robot interaction or on the perception of devices displaying such transcripts: robots, phones, computers, etc. While some research has begun to explore the role of live transcripts in human–human interaction (Echenique et al., 2014; Gao et al., 2014; Yao et al., 2011), informational configurations involving ASR transcripts displayed by robots or voice agents remain largely unexamined. We could not find existing works applying this line of inquiry to smartphone vocal assistants (Siri, Google Assistant, Bixby, etc.,)—which also display transcripts of users’ utterances—or to any recent voice-based conversational agent relying on generative AI that provides transcripts in real time.

### 3.1 Errors and mistakes in human–robot interaction

A rich body of literature directly or indirectly addresses the issue of categorizing types of “errors” or “mistakes” produced by robots during an activity (Honig and Oron-Gilad, 2018; Tian and Oviatt, 2021). This literature overwhelmingly adopts an “etic”, exogenous, point of view on the notion of “errors” or “mistakes”: “errors” are categorized as such by the researcher, independently of whether and how they emerged as errors for the participants themselves while they were involved in a situated activity with a robot (Honig and Oron-Gilad, 2018). This perspective facilitates the subsequent evaluation of the impact of robots’ “failures” on the interaction itself (e.g., Gehle et al., 2015; Schütte et al., 2017), on human behavior (e.g., Giuliani et al., 2015; Hayes et al., 2016; Mirnig et al., 2015; 2017), or on perceptions of the robot (e.g., Ragni et al., 2016; Rossi et al., 2017; Salem et al., 2013; 2015; Tolmeijer et al., 2020). Nevertheless, identifying what constitutes an “error” from the point of view of participants is a challenge that must be regularly addressed even by works that do not specifically focus on this question (Tian and Oviatt, 2021). For example, it is a crucial criterion when asking annotators to code video interactions involving robots (Giuliani et al., 2015). Indeed, a behavior from a robot that constitutes an error from a technical point of view may be treated as entirely appropriate by the study participants; if unaddressed, this distinction may inadvertently become embedded in the coded data (Giuliani et al., 2015).

A branch of research within this literature on errors has extensively explored how a robot can detect that humans are treating its behavior as erroneous; for example, by examining multimodal cues produced by humans when a robot “makes a mistake” (e.g., Bremers et al., 2023; Giuliani et al., 2015; Hayes et al., 2016; Mirnig et al., 2015; 2017; Short et al., 2010). Such robot errors are sometimes intentionally introduced within an experimental context (Centeio Jorge et al., 2023; Mirnig et al., 2017) or observed when they occur in an unplanned way (Giuliani et al., 2015; Mirnig et al., 2015). Through these insights, these works contribute to the more general endeavor of clarifying relevant elements of a situation (including subtle multimodal human behaviors–Vinciarelli et al., 2009) that a robot must perceive and consider to respond adequately (Loth et al., 2015). That is, these investigations of “social signals” attempt to identify, among the potentially infinite number of properties of a setting that can be perceived and attended to, those relevant for the task at hand. Thus, a central contribution of the present article is an inversion of this perspective from which HRI studies are commonly conducted. Rather than focusing on what a robot must detect in human behavior to recognize when it “made a mistake”, we explore the resources (provided by the robot or by the broader setting) that humans mobilize to construct robot behavior as an “error”.

### 3.2 Studies on transparency

This research on the emergence of errors connects with the “transparency” framework, which encompasses recent investigations into what aspects of their internal functioning robots should display (Chen, 2022; Fischer et al., 2018; Kong et al., 2024; Lee et al., 2023; Rossi and Rossi, 2024; Wortham and Theodorou, 2017). In this body of work, transparency is defined as “the visibility of underlying processes leading to a reduction of ambiguity regarding a behavior” (Bečková et al., 2024). “Transparency” is therefore distinguished from “explainability” (Verhagen et al., 2021; 2022), with the former emphasizing which aspects of a robot’s processing are revealed to users (i.e., “what” the robot is doing) and the latter focusing on the reasons behind the robot’s actions (i.e., “why” the robot is doing it—Verhagen et al., 2021).

Within this framework, several studies have explored the perception of humanoid or “social” robots depending on which properties of these robots’ internal functioning were made publicly available in real time (Bečková et al., 2024; Fischer et al., 2018; Frijns et al., 2024; Mellmann et al., 2024). Most notably in regard to the informational configuration we will examine, K. Fischer et al. (2018) studied a medical robot that described its “current and upcoming actions” (e.g., “I am going to come closer now”). They found a positive impact of this type of transparency on participants’ perceived comfort and trust in the robot. However, in a subsequent work on the same robot, Fischer (2018) suggested that “revealing the robot’s real capabilities and underlying processing may hinder, rather than improve, interaction” as “it is especially the implicitness of information that makes human interactions so smooth and seamless”. Mellmann et al. (2024) examined whether displaying a robot’s goals and behavior impacted participants’ perception of its intelligence, anthropomorphism, and agency. Their experimental setup used the Pepper robot’s tablet: in one condition, participants interacted with a Pepper robot whose tablet provided a representation of the robot’s perception of its environment, goals, and current actions (transparency condition). In another condition, participants interacted with a Pepper robot that did not display anything on its tablet (non-transparency condition). Several of Mellmann et al. (2024) findings lean in favor of higher transparency; however, the communication skills of the robot were reported as higher when the robot was not “transparent”.

Overall, this recent body of work on transparency (only briefly outlined in this section) thoroughly analyzes the impact of different degrees of transparency on the way a social robot is perceived by humans (e.g., Angelopoulos et al., 2024; Frijns et al., 2024; Mellmann et al., 2024; Straten et al., 2020). Yet, to date, no study has provided a micro-analytic account of how publicly available transcripts emerge as a significant feature of situated human–robot interaction—if indeed they do. Specifically, one potential pragmatic consequence of social robots’ transparency remains to be explored: its role as a resource on which humans may rely as they ascribe (relevant or inadequate) actions to the robot’s conduct.

## 4 Experimental design

### 4.1 Setup

#### 4.1.1 Naturalistic and controlled data collections

**FIGURE 2:**
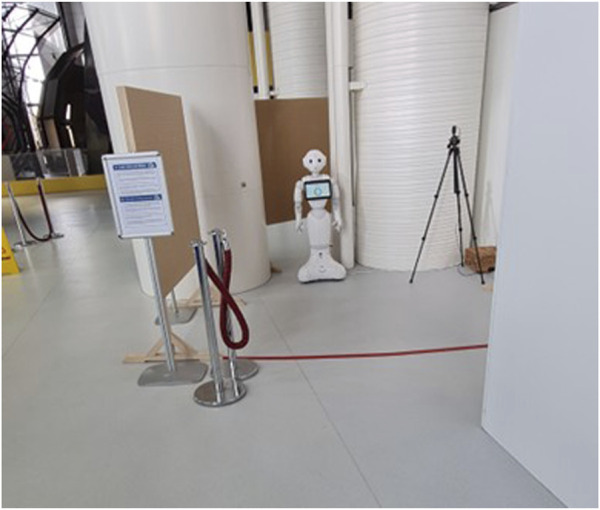
Naturalistic data collection setup for the Pepper robot (loading its chatbot), including an RGPD-compliant sign (left), a red stripe on the ground, and a camera.

To investigate the pragmatic impact of the ASR transcript in situated interactions, we base our analysis on our video corpus recorded in July 2022, Paris, at the Cité des Sciences et de l'Industrie, one of the biggest science museums in Europe. This dataset was collected in accordance with the General Data Protection Regulation (GDPR). Ethics approval was obtained from Paris-Saclay Université Research Ethics Board. The first half of this corpus features 100 naturally occurring interactions with a Pepper robot placed in the hall of the Cité des Sciences et de l’Industrie museum (see Figure 2). The second half of this corpus is composed of 108 controlled interactions that occurred with Pepper in a laboratory open to the public at the Cité des Sciences et de l’Industrie, where participants were asked to wear an eye-tracking device. In both cases, participants (groups or single individuals) interacted with the same Pepper robot, whose speech was generated by a chatbot designed to “converse” on a wide variety of topics. The two video fragments examined in this work (see Section 7) were recorded during our controlled data collection; however, they constitute exemplars of typical orientations to the ASR transcript identified in an analysis of both the naturalistic and experimental parts of our corpus.

#### 4.1.2 Instructions

For the naturalistic data collection, visitors were not briefed by an experimenter before speaking with the Pepper robot. Only the two signs present on both sides of the setup informed participants that this was an experiment focused on “human–robot interactions” and provided information in accordance with the GDPR. Participants were given no indication regarding the robots’ abilities (speaking, hearing, seeing, etc.), nor about the meaning of the text displayed on the tablet (i.e., the ASR transcript).

For the controlled data collection, participants were asked to “speak with the robot in the room for 5 min maximum, and 2 min minimum”. They were not given explanations regarding the conversational design of the robot, the meaning of the ASR transcript displayed on the tablet, or the robot’s abilities. Before entering the room, visitors or their legal guardians were briefed, then asked for their written consent to participate in the experiment and, separately, for the use of the video data in which they would appear. Participants were then asked to wear one pair of eye-tracking glasses (Tobii Pro Glasses 3), which were configured before they started speaking to the robot. In each interaction, only one participant wore eye-tracking glasses. The following analysis of the interactional consequences of the ASR transcript is therefore based on leisurely conversational interactions, i.e., encounters where *no task was pre-defined besides the conversation itself.*

### 4.2 Automatic speech recognition transcript design

Upon hearing a human speak (or a human-like sound), the robot attempted to recognize which words were uttered. To do so, it used both a local Automatic Speech Recognition system and a remote one^5^. If any of these services returned a text input that matched one of the possible answers for the robot’s chatbot, the robot synthesized this answer. Any utterance produced by a human followed by a silence of more than 200 ms was considered complete, transcribed on the robot’s tablet for 3 s (see Figure 1), and responded to by the robot. In all recent Pepper robot models, this 200 ms silence threshold is the default setting used to determine when a user has finished speaking. Similarly, displaying the ASR transcript for 3 s is the default setting in commercial versions of the Pepper robot. We retained these values to ensure our findings remain relevant to Pepper robots currently in use.

After they produced an utterance, participants encountered one of the following behaviors from the robot:

- When the robot was provided with a result from the ASR, and could match a response (in its chatbot) with this specific ASR result, then the robot displayed the ASR transcript on the tablet and triggered its verbal and gestural answer at the same time.
- When the robot was provided with a result from the ASR but had no answer to this specific utterance, then the robot displayed the ASR transcript but indicated verbally that it did not know how to respond.
- When the robot was not provided with a result from the ASR for the sound it received, then the robot displayed a question mark “?” on the tablet and produced an open-class repair initiator (Drew, 1997): “Huh?”, “What?”, “Pardon?”, “Excuse me?”, etc.

Except for the ASR transcripts, the tablet was blank. Notably, these transcribed words displayed on the tablet were the sole basis on which the robot constructed a response to what a human had just said. Through this ASR transcript, participants had full access to the information from the outside world that the robot used to generate its verbal response—regardless of whether they oriented to the ASR in this way. No other information (gestures, tone, appearance of the interlocutor, etc.) was relied upon by the robot.

### 4.3 Robot’s conversational design

The robot’s behavior (speech and gestures) was handled by a simple rule-based chatbot, locally installed on its tablet. The robot did not generate its answers from a language model, nor was it provided with the answers by an external API. However, the robot’s responses covered a wide range of domains. Its chatbot could produce a variety of gestural and verbal responses on 70 widely defined topics: e.g., geography, sports, animals, personal information about the robot, which movement it could do, which songs it could sing, etc. The robot’s software and conversational design were the same in the naturalistic and experimental data collections.

## 5 Methods

### 5.1 Ethnomethodological conversation analysis

The analytical perspective underpinning this research is Ethnomethodological Conversation Analysis (Mondada et al., 2020), hereafter EMCA. Researchers using this micro-analytic approach carefully examine how participants in a setting publicly produce and ascribe actions through the sequential (Sacks, 1995) organization of their talk, gestures, gaze, and overall bodily conduct (Goodwin, 1981; Mondada, 2016). A core tenet of EMCA is its emic orientation: the analysis remains anchored to what participants themselves demonstrably treat as relevant within the interaction rather than imposing external categories or assumptions (Mondada, 2018). Participants’ actions are understood as being produced in a way that makes their meaning available to co-participants, who, in turn, display their own understanding through their subsequent actions (Garfinkel, 1967; vom Lehn, 2019). By attending to this publicly accomplished order of interaction (Schegloff, 2007), EMCA researchers describe the practices through which participants render intelligible to one another what they are doing, and what they take others to be doing. This provides a robust foundation for analyzing, for instance, how a human “user” orients to a feature of a robot’s conduct (e.g., moving its arm) as accomplishing a specific type of action (Kendrick et al., 2020), e.g., as part of a request, a question, a greeting, or even as initiating a handshake. In essence, the EMCA stance treats the relevance of any detail of a setting as *indexical* (Garfinkel, 1967; Mondada, 2018), i.e., as *emergent* from participants’ hearable and observable orientation to the situation, rather than being *a priori* significant. EMCA research routinely involves a rigorous analysis of video-recorded interactions (Heath et al., 2022; Mondada, 2019), allowing for the precise reconstruction (Ten Have, 2007) of how verbal and gestural conduct is coordinated and finely tuned to the setting (e.g., pointing to a specific feature on a technological device at a particular instant in the talk).

### 5.2 EMCA and human–robot interaction

In recent years, “human–robot interaction” has been increasingly examined from the analytical perspective described above: EMCA’s common practices, interests, and concepts have been, so to speak, transposed to situations involving an entity pre-categorized by the researcher as a “robot” (Due, 2023a; Krummheuer, 2015; Pelikan and Broth, 2016; Pitsch et al., 2013; Tuncer et al., 2023). Indeed, this approach is particularly well-suited for investigating how technological artifacts, such as robots, become consequential in situated interaction (Hutchby, 2001)—not by design alone (Lynch, 1995), but through the practical work of participants as they negotiate the intelligibility and relevance of the robot’s conduct moment by moment. A crucial consequence of this “study policy” (Garfinkel, 1967) for human-robot interaction is that technical features of an artifact are only relevant insofar as they are treated as such by participants in the setting being observed by the researcher. In this context, what a robot “does” is not taken as a given and stable fact, but is instead grasped through the (sometimes rapidly shifting—Alač, 2016) orientations of the human interactants towards this entity.

### 5.3 EMCA and ASR transcripts

Ethnomethodological conversation analysis is regularly described as “cognitively agnostic” (Hopper, 2005; Maynard, 2006) or, at least, as uninterested in “the mental processes which go on in the brain when understanding takes place” (Deppermann, 2012). This stance partially results from a basic property of human-human interactions: in typical face-to-face interactions, processes “residing inside the mind of an individual” are not immediately accessible or relevant to co-present participants or to an external researcher. As mentioned previously, these mental processes are not “a discursive phenomenon, which is publicly displayed and collaboratively oriented to by the parties to a conversation” (Deppermann, 2012). To a certain degree, this cognitively agnostic stance extends to robots’ inner processes: it does not matter to the EMCA analyst that the social action that a robot is taken to be doing (by participants *in situ*) was anticipated and programmed by its developers as such an action. For example, when a robot produces a waving gesture with its arm, and human participants respond to this conduct as a greeting wave, it is of little importance for the EMCA analyst whether this robot’s waving gesture (and its triggering condition) was *also* labeled or categorized as a “greeting” in the robot’s programming. Conversely, in this understanding, when a robot publicly transcribes what it hears (and is treated as doing so by co-present interlocutors), the direct phonological receipt (Svennevig, 2004) of what the robot heard becomes “observable-and-reportable” (Garfinkel, 1967) and, therefore, a potentially interactionally relevant property of the setting—both for the EMCA analyst and for co-present participants.

### 5.4 Eye-tracking as a complement to an ethnomethodological and conversation analytic approach

Few studies have currently discussed the potential of eye-tracking as an additional tool for EMCA to pursue one of its core goals of “gaining access to participants’ orientations and perspectives” (Kristiansen and Rasmussen, 2021). A major pitfall identified by Stukenbrock and Dao (2019) and discussed by Kristiansen and Rasmussen (2021) is the indeterminate public relevance, for participants involved in situated interactions, of gaze behavior. Accurate and quantified information about eye fixations, saccades, as well as the first-person perspective of video recordings achieved with eye-tracking glasses, is “specifically unavailable to co-participants and cannot for that very reason have any social significance for the participants in the interaction” (Kristiansen and Rasmussen, 2021). Less intricate gaze behaviors can also be perceptually available to co-participants during an interaction (e.g., someone looking in a specific direction, for a long period or not, etc.) without being responded to as socially meaningful, i.e., as publicly available and reportable phenomena. Hence, eye-tracking, when used as part of a conversational analytic methodology, risks encouraging the analyst to provide *a priori* social relevance (Schegloff, 1993) to all gaze behaviors produced by participants. The analyst’s description of these gaze behaviors (quantified or not) may not reflect co-present participants’ observable orientation to these practices as relevant features of the situation, nor convey what was really perceived by these co-participants while they were immersed in the urgency of a situated interaction.

The previous considerations should not extend to our use of eye-tracking data *as a preliminary step to a qualitative analysis*. Rather than collecting eye-tracking data to bring “new analytic insights” (Kristiansen and Rasmussen, 2021) to an EMCA analysis, we argue that, on the contrary*, an EMCA analysis is fit to bring new insights to purely quantitative eye-tracking results*. That is, it is well-suited to “explain” these data by disclosing which interactional phenomena they aggregate. Hence, we compartmentalized, on one hand, the quantitative overview based on the ASR transcript (Section 6) and, on the other hand, the detailed study of interactional phenomena (Sections 7, 8). Eye-tracking was used as a preliminary tool to get a grasp of whether or not participants focused their attention on the ASR transcript when it appeared. Once it was confirmed that–on average–they did, we explored the *social relevance* of this ASR transcript in constituting the robot’s conduct as a “mistake” or an “error”.

## 6 Gaze data analysis

### 6.1 Conditions

During the experimental data collection, participants (N = 108) were randomly assigned to one of two conditions. We then randomly selected 22 participants from each condition (44 participants in total) for gaze behavior analysis. The total number of participants (44) was based on a practical estimate of how many participants’ gaze fixations could be reasonably mapped, manually verified, and analyzed.

**FIGURE 3:**
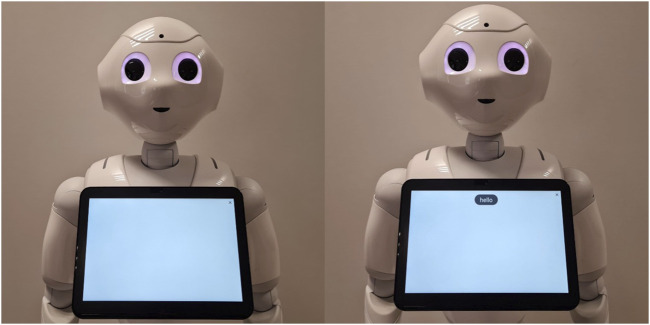
Pepper after hearing “Hello” in the No Transcript condition (left) and in the ASR Transcript condition (right).

- In the “No Transcript” condition (see Figure 3), participants interacted with a robot whose tablet remained completely blank at all times (N = 22).
- In the “ASR Transcript” condition (see Figure 3), participants interacted with a robot displaying an automatic speech recognition (ASR) transcript on its tablet (N = 22).

Aside from the presence or absence of the ASR transcript, the autonomous robot’s behavior was identical in both conditions. On average, the 44 participants conversed with the robot for 4 min and 24 s–that is, for 264 s (SD = 94)^6^. In each of these interaction recordings, only the segments relevant to our analysis were thoroughly transcribed in both their spoken and multimodal details (see Section 7).

### 6.2 Data preparation

#### 6.2.1 Timecoding of utterances and mapping of fixations

**FIGURE 4:**
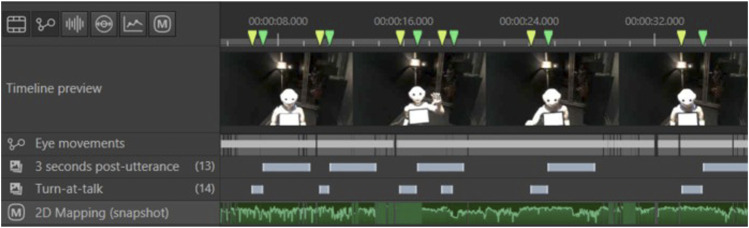
Speaking turns produced by a participant coded as Times of Interest (TOI) on Tobii Pro Lab. The “2D Mapping” layer displays each gaze fixation that was mapped onto a 2D picture of the robot.

For each of the 44 randomly selected participants, we time-coded every moment during which a speaking turn was produced by a human (see Figure 4).

**FIGURE 5:**
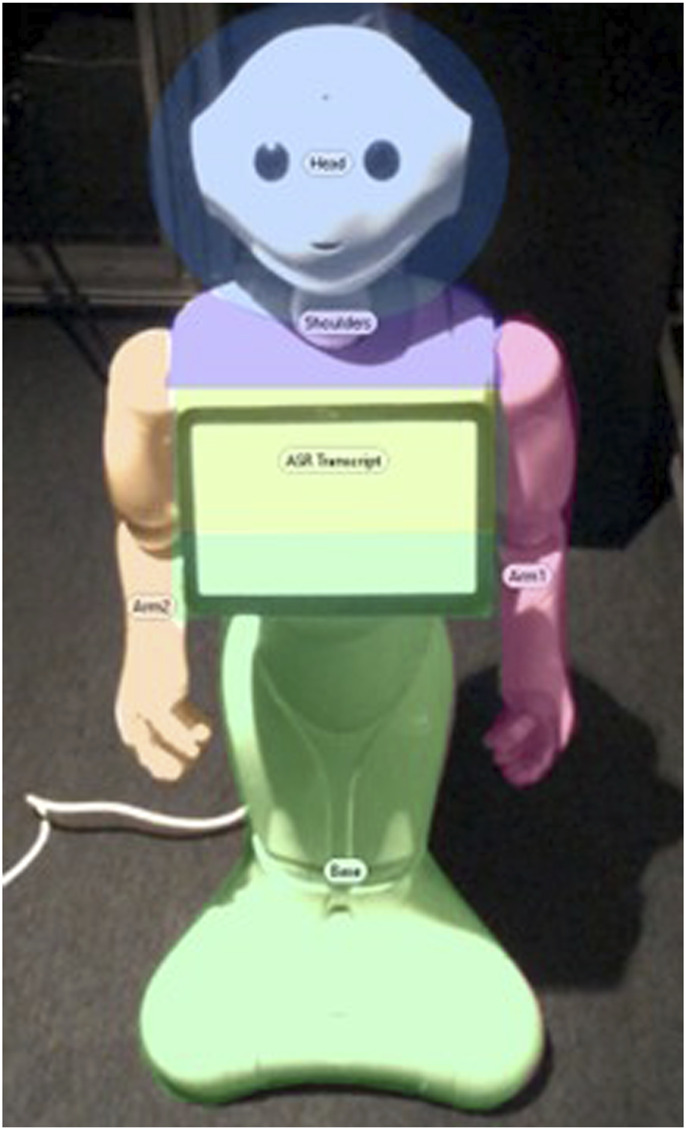
Areas of Interest (AOI) set on a 2D picture of the robot on Tobii Pro Lab.

Meanwhile, a 2D view of the robot was created (see Figure 5). It was divided into several zones:

- Head
- Shoulders
- Arms
- Base
- ASR Transcript Zone (top of the robot’s tablet)

We combined zones 1, 2, 3, and 4 under the category “Body and Head”, to compare them with zone 5 “ASR Transcript Zone” (the top of the robot’s tablet, where the ASR transcript appeared in the ASR Transcript condition). Each gaze fixation from participants was then mapped onto this 2D view^7^ (see Figure 4). This provided us with a distribution of participants’ fixations over the robot’s visible features.

#### 6.2.2 Isolation of periods during which the ASR transcript was visible

In the ASR Transcript condition, the ASR transcript was displayed exclusively over a period of 3 s after the robot heard a speaking turn. The tablet was blank the rest of the time. Hence, in the ASR Transcript condition, to focus on participants’ conduct when they could effectively see an ASR transcript in front of them (and not when the tablet was blank), we extracted participants’ gaze attention during this 3-s post-utterance phase. To facilitate a meaningful comparison (see Section 6.3), we then also extracted this post-utterance phase for the No Transcript condition. In both conditions, this phase of 3 s is especially interesting for conversational-analytic concerns: it corresponds, first, to the display of the ASR transcript (in one condition) but also to the start of the robot’s response to what was said (in both conditions). In other words, *it is the moment where participants can produce an early interpretation of whether the robot properly heard and understood them, either using the ASR transcript or the robot’s verbal and gestural response.*

**FIGURE 6:**
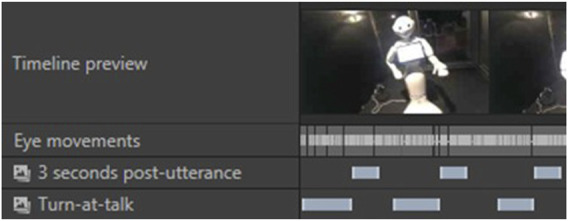
Example of timecoded utterances and post-utterance phases for a human participant.

An additional difficulty was that the number of turns produced by participants varied greatly depending on how long they spoke to the robot: some “conversed” with the robot for a long time, while others spoke for only a few minutes. Hence, for each participant, we considered their gaze behavior during the 3 s that followed each of the *first 20 speaking turns they addressed to the robot* (see Figure 6). In other words, in the ASR Transcript condition, we studied participants’ gaze behavior during the first 60 s (3 s post-utterance multiplied by their first 20 speaking turns) that they spent in front of a (mis)transcription of what they said. In the No Transcript condition, we studied the same post-utterance period except that, in this condition, participants were not provided with a transcript.

#### 6.2.3 Binning eye-tracking data in fixed-duration time intervals

After extracting the cumulated 60 s of “post-utterance” time for each participant, we binned^8^ them in 50 ms interval bins (for a total of 1,200 bins per participant). Then, for each 50 ms bin, we checked how much of this time was spent fixating on each part of the robot. This allowed us to plot the evolution of participants’ average total fixation time (per bin) on the “ASR Transcript Zone” compared to the rest of the robot (see Section 6.4). That is, these steps served as a preparatory stage for the temporal and regression analyses conducted subsequently: they ensured that the descriptive and inferential statistics rely on the same pre-processed data set.

Segmenting gaze activity (or the overall embodied course of action of participants) into 50 ms bins is widely used in gaze research (e.g., Andrist et al., 2015; Ito and Knoeferle, 2022; Klein Selle et al., 2021; Orenes et al., 2019; Venker and Kover, 2015). Although these concerns ultimately proved not to be directly relevant to the analyses presented in this study, this resolution of 50 ms is fine enough to capture the quick timing of gaze coordination reported in conversation-analytic work^9^, yet coarse enough to smooth tracker jitter and minor sampling-rate differences. Accordingly, because 50 ms bins are routinely adopted in eye-tracking research, we defined our bin width *a priori* in alignment with this common practice. Additionally, the shorter bins facilitated the generation of more readable visualizations of gaze evolution over time (see Figure 9).

### 6.3 Results

#### 6.3.1 ASR transcript condition (N = 22)

**FIGURE 7:**
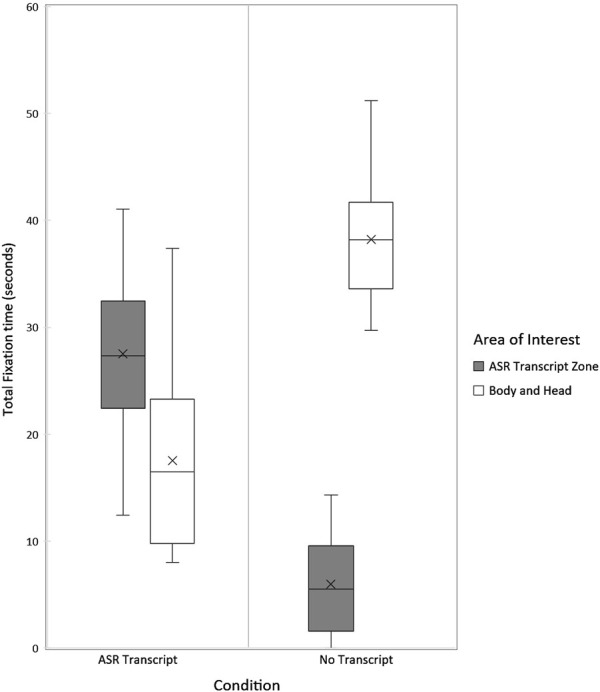
Distribution of participants’ total fixation time on each area of interest in the No Transcript (N = 22) and ASR Transcript (N = 22) conditions, measured during the 3 s immediately following each human speaking turn.

In the ASR Transcript condition, during the 3 s that followed their utterances, participants’ gaze was mostly directed towards the tablet (see Figure 7). On average, after 20 speaking turns, the average total fixation time per area of interest was:

- 27 s for the “ASR Transcript Zone”
- 18 s for the robot’s “Body and Head”.

**FIGURE 8:**
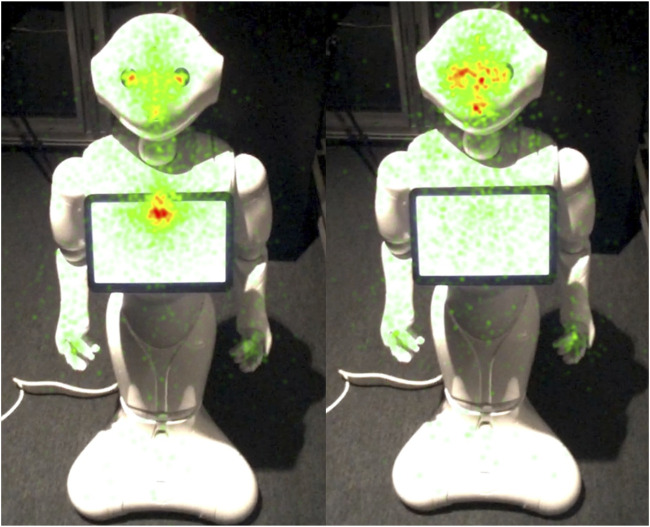
Heatmap of participants’ fixations after they finished their speaking turn in the ASR Transcript condition (N = 22), left, and in the No Transcript condition (N = 22), right.

That is, 61% of the time participants spent fixating on the robot after they finished speaking was focused on the “ASR Transcript Zone” and not on the robot’s gestures or face. The heatmap of participants’ fixations similarly illustrates the high number of fixations on the “ASR Transcript Zone” during this condition (see Figure 8).

The gap between the average time that participants spent gazing at the robot’s “Body and Head” and “ASR Transcript Zone” is confirmed by a paired samples t-test. There is a significant difference in mean fixation time between these two parts of the robot, t(10) = 3.22, p = 0.004. Cohen’s d indicates a medium to large effect size (Cohen’s d = 0.70). Data is normally distributed (Shapiro-Wilk Test p = 0.5374).

#### 6.3.2 No transcript condition (N = 22)

In the No Transcript condition, participants’ gaze was mostly directed towards the robot’s “Body and Head” during the 3 s that followed their first 20 utterances (see Figure 7) and barely towards the “ASR Transcript Zone” (which remained blank). On average, after 20 speaking turns, the total fixation time per area of interest was:

- 6 s for the “ASR Transcript Zone”
- 38 s for the robot’s “Body and Head”.

The difference between the average time that participants spent gazing at the robot’s “Body and Head” and the “ASR Transcript Zone” is confirmed by a paired samples t-test. There is a significant difference between the mean total time during which participants fixated on these two parts of the robot (t(21) = −18.43, p < 0.0001). Cohen’s d indicates a large effect size (d = 3.93). Shapiro-Wilk Test suggests that the data is normally distributed (p = 0.3044). This relative absence of focus on the tablet is also apparent in the heatmap of participants’ fixations after they finished their speaking turn in the No Transcript condition (see Figure 8).

### 6.4 Evolution of post-utterance gaze fixations over the course of the interaction

Because the time participants spent looking at the “ASR Transcript Zone” (top of the robot’s tablet) was minimal in the No Transcript condition, we then focused exclusively on the evolution of participants’ gaze attention during the ASR Transcript condition. That is, we sought to determine if participants changed their gaze behavior over the course of an interaction where they faced a transcript of what they said.

**FIGURE 9:**
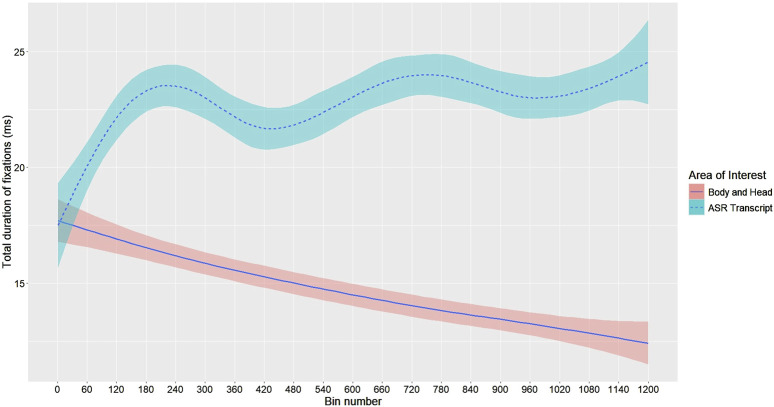
Post-utterance fixations over time by area of interest (N = 22). The x-axis is divided into 50 ms bins; a major tick every 60 bins (i.e., 3 s) marks the start of a new “post-utterance phase”—i.e., the 3-s period following each participant’s utterance during which the ASR transcript was visible. The plot therefore displays 20 successive 3-s post-utterance phases concatenated in time order, while the speaking turns between these post-utterance phases are omitted. Solid (ASR Transcript) and dashed (Body and Head) lines show the GAM-smoothed mean total fixation duration per bin. The shaded bands around each line indicate ±1 standard error (SE) of these means.

We plotted the evolution of participants’ average time spent fixating on the “ASR Transcript Zone” (versus the rest of the robot) over their first 60 s of “post-utterance” time (see Section 6.2.3). The results suggest that, in the ASR transcript condition, participants’ gaze attention *gradually focused on the “ASR Transcript Zone” when they finished speaking* (see Figure 9). Conversely, there was a decrease in their gaze attention towards the rest of the robot’s body (head, hands, etc.) during this period. In other words, over the course of their first 20 turns at talk, participants seemed to increasingly fixate on the “ASR Transcript Zone” after each utterance. Note that, to enhance readability and better visualize trends, fixation durations in Figure 9 were smoothed using a generalized additive model (GAM) with a cubic regression spline basis (see footnote 8 for the script used to generate Figure 9). However, all statistical analyses were conducted on the unsmoothed data.

To verify this observed trend, we conducted a linear regression analysis with the bin number (representing consecutive 50 ms intervals^10^) as the predictor and the mean gaze duration as the outcome. The regression model was significant, F(1,1198) = 18.37, p < 0.001, explaining approximately 1.5% of the variance in gaze duration (adjusted R^2^ = 0.014). For every 50 ms increase in time (one bin), participants gazed at the “ASR Transcript Zone” for an additional 0.0023 ms on average: b = 0.0023, t(1198) = 4.286, p < 0.001.

A complementary way of investigating the progressive focus of participants’ gaze on the “ASR Transcript Zone” was to analyze the difference between the time they spent fixating on this area and the time they spent fixating on the rest of the robot. That is, to study the *relative* focus of participants’ gaze attention between both areas of interest (since they could also look elsewhere in the room) over time. Hence, we calculated the difference in mean fixation time between the “ASR Transcript Zone” and the “Body and Head” of the robot for each 50 ms interval (i.e., for each bin)^11^. We then conducted a linear regression analysis with the bin number as the predictor and this difference as the outcome. The regression model was significant, F(1,1198) = 48.61, p < 0.001, explaining approximately 3.9% of the variance in the gaze difference (adjusted R^2^ = 0.038). For every 50 ms increase in time (one bin), the difference in gaze duration between the “ASR Transcript Zone” and the “Body and Head” increased by 0.0066 ms on average: b = 0.0066, t(1198) = 6.972, p < 0.001. Hence, the post-utterance gradual focus on the “ASR Transcript Zone” is statistically significant but minimal: the model’s R^2^ value suggests that the bin number (i.e., each passing 50 ms) explains only a small portion of the variance in gaze time.

Note that, for the previous time-trend analyses, we first averaged fixation duration across all participants within each 50 ms bin (bin n°1 = 0–50 ms… up to bin n°1,200 = 59,950–60,000 ms). This produced a single series of 1,200 mean values that served as the outcome variable in the linear-regression tests reported above. In other words, for the previous linear regressions, *the unit of analysis was the individual time bin, aggregated across all participants* ^12^.

- For the first regression (“ASR Transcript Zone” only trend), the y-values were the mean fixation durations on the “ASR Transcript Zone” for each bin (1–1,200), and the x-values were the bin numbers.
- For the second regression (“Tablet” vs. “Body and Head” difference), the y-values were the difference in mean fixation durations between the “ASR Transcript Zone” and the “Body and Head” of the robot, averaged across participants per bin. The x-values were again the bin numbers.

### 6.5 Discussion: gaze analysis as a preliminary overview

The analysis of participants’ gaze behavior provides three main findings:

- When an ASR transcript was displayed, participants gazed at it more than any other element of the robot or of the setting, after they completed a speaking turn.
- This post-utterance focus on the ASR transcript very slightly increased during the interaction at the expense of other parts of the robot (its gestures, its head movements, etc.). After they finished speaking, and while the robot started to respond verbally and gesturally, participants gazed more and more at the robot’s tablet, and less and less at the robot’s head or body.
- When no transcript was displayed (when the robot’s tablet remained blank), participants barely gazed at the tablet.

The previous observations are significant on their own. Yet, this pronounced focus on the transcript begs the question of its *local relevance* (as a resource, as a remarkable phenomenon, etc.) in situated interaction. That participants gazed at the transcript more than the rest of the robot does not entail that this transcript was, locally, treated as a publicly available and accountable (Kristiansen and Rasmussen, 2021) “conduct” of the robot—the situational relevance of the ASR transcript is not demonstrable by solely describing how long it was looked at. After the previous statistical summary, participants’ gaze attention on the ASR transcript is therefore entirely left to be “explained” in an interactional sense by describing how and for which activity this transcript was practically used (if it was) in local contexts. This is where a micro-analytic EMCA perspective comes into play, to grasp the endogenous organization of interaction of which the ASR transcript was a potentially relevant feature^13^. Through the detailed analysis of qualitative fragments, we attempt to highlight the local interactional phenomena aggregated in the quantitative overview we have first produced. For example, was the ASR consequential in how certain actions were “ascribed” (Levinson, 2012) to the robot, once it started to respond? Was the ASR transcript indexed by participants during the repair sequences they initiated? An EMCA approach appears fit to clarify the typical interactional practices in which took place the “attention economy” objectified by the previous eye-tracking analysis.

## 7 Qualitative fragments

The following fragments were selected from our corpus for three reasons:

1. They are exemplar cases. They display, in an acute form, a common orientation towards the ASR transcript that partially explains (from an emic perspective) the gaze data detailed above. That is, they bring into view some recurring practices that played out behind our quantitative results: i.e., typical practices (indexing the ASR transcript) observable during the interactions that took place in the previously described experiment. In our experimental data, in the condition where the ASR was present, 23 interactions (out of 54 recorded) contained at least one demonstrable reference to the ASR transcript by a participant; often several times within the same interaction. In particular, 9 of these 23 interactions contained at least one instance of the phenomenon we point to in the following excerpts: namely, the retrospective re-evaluation of the robot’s action relying on the ASR transcript^14^.
2. They feature (what is treated by participants as) mishearings or misunderstandings from the robot. As such, these troublesome exchanges are more likely to reveal participants’ orientation towards the ASR transcript compared to perfectly smooth interactions (Förster et al., 2023). Because “it is hard to determine how something works when we only see it functioning unproblematically” (Stivers et al., 2023), studying “when things go wrong” is a typical strategy to uncover the seen-but-unnoticed methods through which humans accomplish their daily activities ever since Garfinkel’s breaching experiments (Garfinkel, 1967). Limited or complete breakdowns in an interaction provide opportunities for participants to publicly display and reconstruct their interpretation of the ongoing situation (Due, 2023b; Garfinkel, 2002).
3. They display “re-evaluations” of an action previously ascribed to the robot. These fragments were selected as cases in which the meaning of the robot’s conduct is hearably negotiated between interlocutors. As mentioned above, many participants explicitly relied on the ASR transcript as a resource in (publicly and visibly) ascribing an action to the robot; the following fragments are especially clear examples of such cases. In a different vocabulary, these interactions involving “re-evaluations” of the robot’s actions functioned as “perspicuous settings” (Garfinkel, 2002). In particular, they allowed an observer to grasp what action the robot was initially ascribed by a participant before the ASR transcript was introduced as a relevant parameter of the setting by another participant.

These fragments were transcribed following Mondada’s multimodal transcription conventions for embodied conduct (Mondada, 2016) and Jefferson’s conventions for verbal conduct (Jefferson, 2004). Transcription conventions are provided at the end of this paper. In each fragment, PA1 is the participant wearing the eye-tracking glasses.

### 7.1 The ASR transcript as a resource for third parties in contesting the relevance of (embodied or verbal) responses from the robot

#### 7.1.1 Fragment 1: using the ASR transcript in contesting the definition of the situation as one of intersubjectivity

**FIGURE 10:**
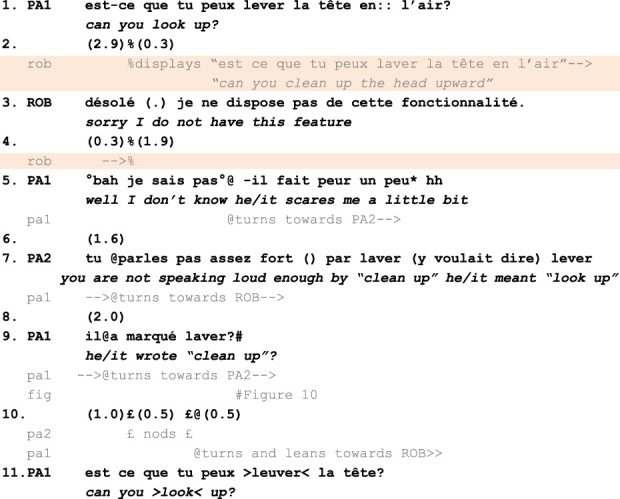
Fragment 1. Multimodal transcript of participants’ conduct following Mondada’s and Jefferson’s conventions. Bold lines represent participants’ talk, while the lines immediately below capture embodied conduct. Content displayed on the robot’s tablet is highlighted in orange. This fragment is described in detail in the following subsection.

The first fragment is presented below (Figure 10).

#### 7.1.2 Analysis

The fragment starts with the rejection of PA1’s request (L.1) by ROB. Even though ROB’s utterance denies what PA1 was asking for, it is not made relevant as an inadequate second pair part. In other words, although ROB’s utterance halts the activity at hand by rejecting (L.3) PA1’s request, *it is a sequentially relevant response, of the type and form made relevant by PA1’s request.* PA1 then produces a complaint to PA2 about being scared of ROB (L.5). Doing so, she creates an answer slot for PA2. However, PA2 does not use this available slot to answer PA1’s complaint but, instead, to produce an alternative account of the interaction that just unfolded. He points out (L.7) that ROB heard “laver” (“cleaning”) instead of “lever” (“looking up”) and provides a possible cause for ROB’s mishearing (“you do not speak loud enough”, L.7). Significantly, in doing so, PA2 indexes the content of the ASR transcript (L.7) a few seconds after this transcript disappeared from ROB’s tablet (L.4). This delay indicates that PA2 has read the ASR transcript *before* a form of trouble was publicly manifested by PA1 (L.5) and not only *in reaction* to such trouble.

**FIGURE 11:**
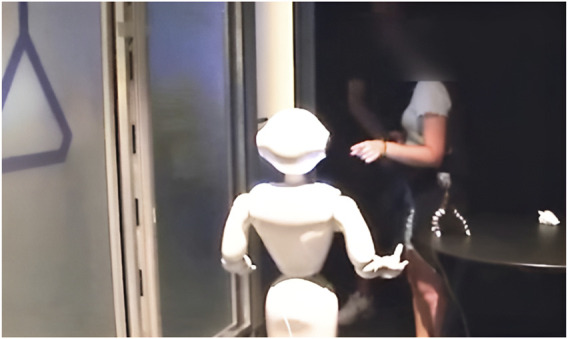
PA1 points at ROB’s tablet while asking PA2 for confirmation about what it previously displayed.

Once details about what was written on the ASR transcript are provided to PA1 by PA2, this information completely reconfigures, *a posteriori*, the action attributed to ROB by PA1. By asking a confirmation question, “it wrote clean up?” (L.9, Figure 11), PA1 manifests she is dealing with new properties of the situation, on which she did not previously rely to interpret ROB’s conduct. This reframing is observable when, after this turn, PA1 immediately casts the robot’s previous answer as inadequate by repeating her own previous question (L.11) while insisting on the term which was stated, by PA2, to be mistranscribed (“can you > look < up?”). This re-evaluation of ROB’s conduct illustrates how much the social significance of verbal and embodied responses from the robot can be contingent upon what the robot transcribes on its tablet.

Crucially, here, this re-evaluation is not about the *type* of response that ROB does. In this fragment, ROB’s conduct rejects a request: at this level of description, on the surface of the interaction, ROB produces an adequate second pair part. However, this action (of refusing a request) is re-evaluated as *indexed to the wrong speaking turn.* Therefore, what is revised here is not the “action” in a generic sense. Rather, what is reconsidered is this action as activity-relevant, i.e., its status as a response to what was really requested by human participants. ROB’s response is not problematized as a response to a request but *as a response to a specific request.*

Finally, although in this case it is the bystander who monitored and verbalized the robot’s mistranscription of the main speaker’s utterance, referencing the ASR transcript is not exclusively a bystander’s role in our corpus of multiparty interactions. In the condition where the ASR transcript was available, 23 out of 54 recorded interactions featured at least one demonstrable reference to the transcript by a participant (See Section 7), often multiple times within a single interaction. In 8 of these cases, a bystander initiated at least one speaking turn referring to the transcript. The fragment selected here was chosen for reasons of analytical clarity: the bystander’s denial of the main speaker’s interpretation allows us to grasp in minute detail how participants negotiated the issue of “what action the robot had been performing.” This case aligns with prior research documenting the consequentiality of bystanders’ contributions in multiparty human–robot interactions (see, e.g., Krummheuer, 2015; Pitsch, 2020; Rudaz and Licoppe, 2024).

#### 7.1.3 Fragment 2: using the ASR transcript in accounting for a breakdown in interaction after it arose

**FIGURE 12:**
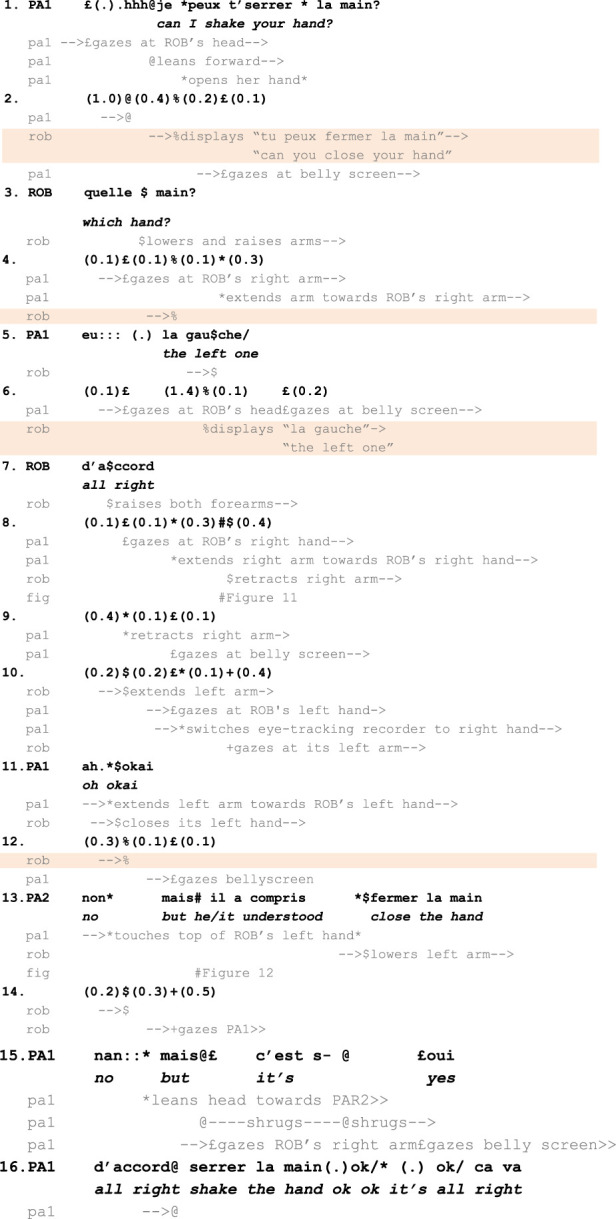
Fragment 2. Multimodal transcript of participants’ conduct following Mondada’s and Jefferson’s conventions. Bold lines represent participants’ talk, while the lines immediately below capture embodied conduct. Content displayed on the robot’s tablet is highlighted in orange. This fragment is described in detail in the following subsection.

The second fragment is presented below (Figure 12).

#### 7.1.4 Analysis

**FIGURE 13:**
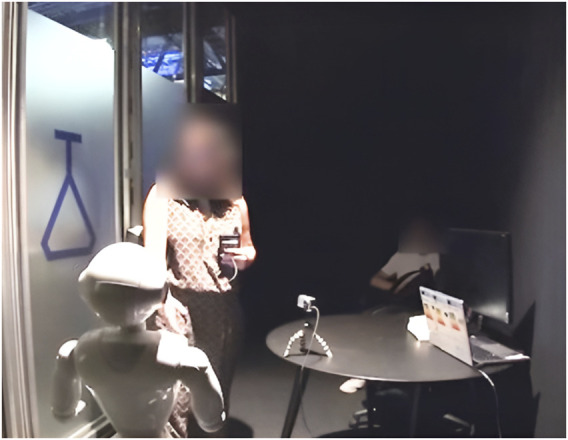
PA1 extends her hand towards ROB’s right hand.

**FIGURE 14:**
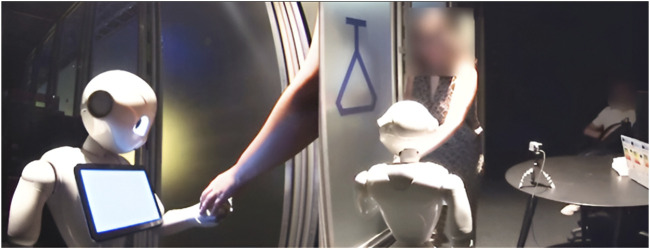
PA1 touches ROB’s left hand while PA2 states that ROB understood “close the hand”. The two photos are different angles of the same moment.

This fragment illustrates a nominal use of the ASR transcript as a resource to identify, account for, or repair trouble in interaction. From a purely technical point of view, it begins with a speech recognition error by the robot, which displays “can you close your hand” on its tablet, although PA1 asked “can I shake your hand?”. PA1 interprets ROB’s subsequent left arm gesture as the initiation of a handshake (L.8, Figure 13), reconsiders this course of action when ROB lowers its left arm and raises its right one, frees her right hand from the object she was holding (L.10), and is finally confronted with ROB’s closed hand when she approaches her right hand (L.11 and L.13, Figure 14). After this long sequence of visible struggle to coordinate between PA1 and ROB, PA2 produces an account of ROB’s understanding of the situation (L.13). Over the course of the experiment, PA2 had regularly monitored the transcripts displayed on the robot’s tablet (not in transcript). However, as this specific scene unfolded between PA1 and ROB, he had remained silent until its completion. Prefacing his account with a “no but” (L.13), he states that the robot “understood ‘close the hand’”, L.13). Doing so, *PA2 demonstrably contests PA1’s embodied interpretation of the interaction (as an ongoing mutual handshake)* and attributes a different cognitive state (of “understanding”) to the robot.

On the interactional surface (independently of the question of whether PA1 is pretending the robot is collaborating to achieve a mutual handshake), this fragment therefore displays two different treatments of what is (for an external observer) the “same” gesture produced by the robot. The first treatment, by PA2, indexes an informational configuration that includes the tablet to contest the sequential relevance of the robot’s conduct. The second one, carried out by PA1, orients to “what is going on” in this situation by (publicly and demonstrably) relying exclusively on the robot’s verbal and gestural conduct. However, PA2 treats these two interpretations as unable to coexist. By prefacing his turn with a “no” (Lerner and Kitzinger, 2019), L.13, PA2 does more than accounting for PA1’s trouble to properly complete her handshake gesture with the robot. PA2’s turn belongs to a category of “no-prefaced rejection of the trouble source” that, following Lerner and Kitzinger (2019), “explicitly disallow[s] the trouble source as having been mistaken”. That is, PA2 negates that a mutually understood handshake was ever present, i.e., that the apparently coordinated gestures of ROB and PA1 manifested an intersubjectively shared reality between them. In other words, in PA2’s interpretation, *the action that ROB achieved by raising its hand partly depended on what was written on its tablet* and not only on the relevance of the robot’s gesture as a potentially adequate response to PA1’s request. The ASR transcript allows PA2 to clarify as inadequate the second pair part produced by the robot (here, extending and closing its left hand) that could, otherwise, be treated as sequentially relevant, as PA1 was doing until then. The public availability of the ASR was central in the re-evaluation of the situation as a misunderstanding rather than as a (non-smooth) mutually coordinated handshake.

## 8 Discussion: the practical relevance of the ASR transcript

### 8.1 Troublesome ASR transcripts could override the verbal and gestural response of the robot

In the video fragments analyzed above, the robot’s ASR transcript was used as a resource unlike any typically found in ordinary human face-to-face interaction. The resulting informational configuration was consequential in shaping the timing and composition of repairs initiated by human participants. It impacted the terms participants treated as troublesome in previous turns (as words “misheard” or “misunderstood” by the robot, e.g., Fragment 2, L.13) and even which syllables were stressed in their repairs (e.g., Fragment 1, L.11). For the two groups of participants we examined, in the presence of a systematic ASR transcript, what “interacting with a robot” consists of practically was pulled further away from the concrete processes that constitute human–human interaction: the transcript modified the ordinary practices that were (or could be) produced, and the pragmatically relevant properties of the interaction^15^.

Note that the impact of the ASR transcript on participants’ conduct highlights that, from the point of view of the robot itself, considering the ASR transcript is crucial to make sense of the interactions in which it is involved. In fragments analyzed in this work, human participants’ conduct (e.g., the sudden initiation of a repair sequence although the robot’s conduct was treated as relevant so far) is only fully understandable if the presence of an ASR transcript in front of them is taken into account. If a robot were to attempt to interpret participants’ reactions without this crucial contextual element of “what it displayed on its tablet”, it would miss one of the most consequential features of these situated interactions. In other terms, it is critical for the robot to be able to identify when the conduct of human participants (e.g., repairs, accounts, repetitions, etc.,) is not produced in reference to something it said (i.e., it is not an answer to a previous speaking turn) but, rather, in reference to something it is currently displaying. These observations therefore add to the list of key parameters that embodied agents should perceive and consider (Giuliani et al., 2015; Mirnig et al., 2017; Vinciarelli et al., 2009) to better understand what human participants are indexing in their speaking turns–thereby helping the robot to identify when its actions are being framed as a “mistake” by human participants (Frijns et al., 2024; Giuliani et al., 2015; Mirnig et al., 2017).

### 8.2 Impact on the “sequential plasticity” of the robot’s turns

Could it be that some fundamental conditions or encompassing mechanisms of human sociality (Mondémé, 2022) were highlighted or altered by the unusual informational ecology visible in the previous fragments? Garfinkel’s early experiments (Eisenmann et al., 2023; Garfinkel, 1967; Ivarsson and Lindwall, 2023) highlighted the demonstrable tendency of participants to interpret immediately adjacent talk as responsive to their own and as displaying an understanding of their prior conduct. In human-robot interactions, many behaviors from a robot treated by co-present members as a successful second pair part are, from the point of view of the roboticists who programmed this robot, the result of an unforeseen sequence of events in which the robot did not, in fact, adapt to the humans—see, e.g., Pelikan et al. (2020), Rudaz et al. (2023), Rudaz and Licoppe (2024), and Tuncer et al. (2023). When considering this regularly “fortuitous” (Tuncer et al., 2023) character of a robot’s successful responses to humans’ actions, the ASR transcript limited the “sequential plasticity” (Relieu et al., 2020) of the robot’s conduct by clearly indicating—for the numerous participants who treated it that way—what sequence of words the robot was responding to. For example, in Fragments 1 and 2, the ASR transcript contributed to removing the vagueness of the robot’s response “as an answer-to-the-question” (Garfinkel, 1967—see Fragment 1) and “as an answer-to-the-request” (see Fragment 2): the exact phonological form (Svennevig, 2004) of what the robot was responding to was apparent to the main human speaker or, at least, to a bystander.

The resulting informational configuration narrowed down the range of behaviors from the robot which could be directly treated as (adequate) *social actions*—that is, as responsive to the situated interaction and as “making relevant a set of potential next actions” (Tuncer et al., 2022). For example, because the ASR transcript limited the range of meaningful intentional patterns that could be connected with the robot’s observable behavior, such a context offered fewer resources for human participants to “safeguard the robot’s status as an agent” (Pelikan et al., 2022) or to produce other practices—well documented in HRI—through which a robot is ordinarily maintained as a competent interactant by human participants (e.g., Rudaz and Licoppe, 2024). If, in interaction, each action “tests the hypothesis a participant has about a co-participant’s response to her/his action” (vom Lehn, 2019), these “experiments in miniature” (vom Lehn, 2019) took a very different form in these human-robot interactions where the robot featured a transcript of what it “heard”.

### 8.3 Does the ASR transcript enforce a cognitivist definition of mutual understanding?

Based on the previous discussion, the orientation of the previous participants toward the ASR transcript can be described in two ways.

1. A safe manner of verbalizing these participants’ practices is to say that it became a “members’ problem” (Garfinkel, 1967) that the ASR transcript displayed an adequate receipt of their previous turn. The performance that was expected from the robot was not merely gestural and verbal. It was also an “auditory” performance. For all practical purposes, it was relevant for them that the robot displayed a too-dissimilar transcript of what they said.
2. Yet, in the EMCA endeavor to represent as precisely as possible participants’ own emerging categories during their situated activity, another level of description might be more faithful to the orientation displayed by these participants (and by many others in our larger corpus) towards the ASR transcript: they were led to enact a cognitivist definition of mutual understanding. In other words, these participants publicly cared about what was inside the robot’s “head” or “algorithm” when they treated the situation as repairable.

In this second interpretation, as a technological artifact, the ASR transcript facilitated a specific orientation to human–human understanding (in a conversation), which stands at the other end of the spectrum compared to the way it is conceptualized in ethnomethodology and conversation analysis. If, as Suchman (1987) mentions, “[e]very human tool relies upon, and reifies in material form, some underlying conception of the activity that it is designed to support”, the speechbar enforced a definition of “understanding”^16^ as sharing a “similar mental representation about the world” (Albert and Ruiter, 2018) rather than, as it is generally described in conversation analytic works, being “related to the next action achieved by the co-participant” (Mondada, 2011—see also Coulter, 2008; Dingemanse et al., 2023; Mondémé, 2022). It favored an orientation to progressivity in conversation as based on a cognitively shared reality (Searle, 1969), rather than as “being able to ‘go on’ with each other” (Sterponi and Fasulo, 2010). From a different theoretical perspective, the ASR transcript led participants’ “theory of mind” of the robot (Angelopoulos et al., 2024; De Graaf and Malle, 2017; Wortham et al., 2016) to take a central place in their publicly displayed interpretation of the robot’s actions. In particular, the *intention* of the robot (De Graaf and Malle, 2017; Malle and Knobe, 1997) to produce the action it was originally taken to be producing (a handshake, a refusal to a specific request) became a central concern.

For the bystanders in fragments 1 and 2, it did not only matter that the robot produced verbal and gestural answers to what was just said, but *it also mattered that these verbal and gestural answers stemmed from an entity that had properly (displayed to have) heard what had been said*. In order to ascribe actions to that entity, in order to grasp what it was “doing”, an element internal to that entity mattered (what phonological reconstruction it had produced of the previous talk), possibly more than its verbal and gestural response. In other words, displaying the (in)adequate phonological reconstruction of the utterance that a turn responded to regularly impacted the relevance of this turn as an adequate response–it played a key role in participants’ framing of that conduct as a “failure”, a “mistake”, a “misunderstanding”, i.e., as a troublesome behavior. The ASR transcript rendered practically feasible a cognitivist definition of mutual understanding “as a mental process” (Ryle, 1949; Wittgenstein, 1953), i.e., as “grasping what is in the other’s mind” (Shotter, 1996). On this view, participants displayed an orientation towards the existence of a form of shared reality (at least regarding the phonological identification of what they said) as required to progress the interaction. This claim is partially reinforced by the comparison with our participants’ conduct in the second condition of the experiment, where no ASR transcript was available. In this “No Transcript” condition, we found no sequence during which the robots’ exact hearing of what a human participant just said emerged as a necessary condition to continue with the ongoing activity. This is strikingly different from what we examined in the previous video excerpts.

Nevertheless, this interpretation (that participants were led to enact a cognitivist definition of mutual understanding) inevitably goes beyond what our data can substantiate based on participants’ observable and hearable orientations to the setting. Since what is in the robot’s “head” (i.e., what is processed by its algorithms) can only be documented through the ASR transcript displayed on the tablet, it is ultimately impossible to rigorously distinguish a strict orientation to the transcript from an orientation to the robot’s internal states. From a strictly observable standpoint, what can be conclusively demonstrated is that the content displayed on the robot’s tablet was recurrently relevant for participants—for all practical purposes—in progressing or repairing the interaction. In mundane conversation, participants ordinarily do not initiate repair as soon as a pre-existing and spotless state of intersubjectivity starts to deteriorate even in the slightest; they initiate repair when their shared understanding is not sufficient for their “current practical purposes” anymore (Linell and Lindström, 2016; Schutz, 1972). However, in the presence of an ASR transcript, each time “what was heard by the robot” differed from what was publicly said by the human, this information was accessible by said human. Consequently, a minimal reading of our data is that the presence of an ASR transcript constantly threatened to trouble participants’ practical sense of what was a disregardable non-significant mismatch to reciprocal alignment (Shotter, 1996; Sterponi and Fasulo, 2010) or, conversely, what was a locally relevant misunderstanding which should be addressed–even at the cost of a “time out” (Heritage, 2007) in the current course of action (Bolden, 2012; Schegloff et al., 1977).

### 8.4 Implications for design: ASR transcripts and “conversational moves” available to a robot

Some design considerations can be drawn from the empirical observations above. Many typical conversational practices are likely to be difficult to perfectly replicate for a robot that displays transcripts: independently from a conversational agent’s competence or technological advancement, systematically displaying an ASR transcript (in leisurely conversational interactions, rather than heavily task-oriented contexts) unavoidably complicates the accomplishment of “conversational moves” that rely on the absence of an internalist window on “what was heard”.

For example, the conversational move that Liberman (1980) names “gratuitous concurrence” may, *a priori*, be hindered by the informational configuration associated with ASR transcripts. In Liberman’s (1980) definition, gratuitous concurrence is the action of providing an interlocutor with a “confirmation of comprehension” without having comprehended what this interlocutor has said: e.g., when a recipient produces the change-of-state token (Heritage, 1985) “oh” in response to an utterance that they did not understand—as evidenced at a later point in the conversation. Between humans, a useful resource for gratuitous concurrence is therefore the inaccessibility, for the other parties, of “what is being concurred with” in the speaker’s head. In the case of a mishearing, the conversational “move” of gratuitous concurrence (e.g., responding “yes” to a sentence that one did not hear entirely) is therefore not replicable in the same conditions for a robot displaying a transcript, because part of the internal state of the robot is publicly visible. For such a robot, being able to “pass over” an ambiguity about the phonological identification of what the human said (e.g., by laughing although it did not hear what its interlocutor said, by tentatively producing the change-of-state token “oh”, etc.,) would require that the human either does not publicly attend to the ASR transcript as a relevant property of the interaction or orients to it as something else than a transcript.

A design dilemma (as well as a moral dilemma) stems from this state of affairs: should conversational agents prevent misunderstandings at all costs? Or should they act similarly to typical human interlocutors? In sum, do we want conversational agents to be able to use the same conversational “moves” as humans? Beyond questions regarding the ideal “interpretative latitude” between robots and humans to facilitate the progress of the talk, it is worth considering if, for ethical reasons, the robot should be provided the same “tools” (e.g., indeterminacy about what it really heard) as humans to maintain the sequential relevance of its turns and, through this, its perceived conversational competence.

### 8.5 Limitations and implications for future research

The current study examined only one configuration of transcripts: the ASR transcript appeared on a tablet positioned on the robot’s torso, for a fixed duration of 3 s, immediately after the robot detected the end of a human utterance. This design involved a single criterion for triggering the ASR transcript display—namely, the recognition of human speech by the robot. Future research could explore alternative configurations, including:

1. Displaying the transcript only when the ASR confidence score falls below a certain threshold (i.e., when the human is likely to have been “misheard”).
2. Displaying the transcript alongside the corresponding ASR confidence score (to highlight when the robot is likely to have mistranscribed what was just pronounced).
3. Displaying transcripts selectively, based on the type of human turn.

With regard to the third point, a promising alternative would be to display an ASR transcript only after specific kinds of utterances, rather than systematically after every turn. This would build on the parallel between the interactional function of written transcripts and verbal “displays of hearing” (Svennevig, 2004) occasionally produced in human talk, such as repeating one’s interlocutor’s utterance. Indeed, humans do not routinely produce such displays of hearing after every turn of their interlocutor. On the contrary, displays of hearing are accomplished in response to specific local contingencies, where they play a meaningful role in coordinating action. As Svennevig (2004) notes, conversationalists only “sometimes need to display explicitly that they have registered a piece of information”. This “need” arises mainly when an interlocutor’s previous contribution “does not project any further talk to come” and that, as a consequence, the adequate registering of the prior turn cannot be displayed “indirectly in the design of the next relevant action” (Svennevig, 2004): for example, *when a participant is being provided with someone’s name, a date, or a schedule*. A possible design would thus be to display an ASR transcript exclusively after these types of turns^17^.

## 9 Conclusion: vagueness as a core feature of interaction

In the previous fragments, what locally emerged as interactional trouble (a “failure” to co-produce a handshake, a “mishearing” or “misunderstanding” on the robot’s part, etc.,) was not a pre-given. That “something went wrong” in the ongoing activity was not merely discovered by participants: it was publicly displayed and enforced as an accountable phenomenon. In our two examples, this interactional work was carried out by bystanders standing behind the participant speaking directly to the robot: they challenged the main speaker’s visible orientation toward the interaction as requiring no repair. Crucially, in treating the robot’s conduct as inadequate, these bystanders heavily indexed the information provided by the automatic speech recognition transcript—even when the robot’s embodied and verbal conduct had previously been treated as entirely relevant. In sum, while our preliminary quantitative analysis revealed that participants’ gaze frequently focused on the automatic speech recognition transcript in our corpus (see Section 6) at the expense of other parts of the robot, a qualitative micro-analytic approach indicates that, in situated cases, this systematic transcript also served as a pivotal local resource for some participants in publicly accounting for the robot’s conduct as either relevant or irrelevant. From this perspective, following Fischer’s (2018) suggestion, transparency could be re-evaluated in terms of opportunities for action provided to human participants.

Provided the analysis we outlined in this work holds true, the previous fragments exemplify in various ways that attempting “to produce unambiguousness” (Meyer, 2019) can “unnecessarily complicate the practical situation” (Meyer, 2019)^18^. Transcriptions or descriptions are not “views from nowhere” (Nagel, 1989), merely standing next to the action: they are part of the setting, available to be interpreted as constituents of the ongoing action of the participant producing them (Mair et al., 2021). As Garfinkel (1967) mentions, “members’ accounts, of every sort, in all logical modes, with all their uses, and for every method for their assembly are constituent features of the settings they make observable”. When treated as such (i.e., when interpreted by participants as “displays of hearing”—Svennevig, 2004), automatic speech recognition transcripts impacted what actions were ascribed to the robot (Levinson, 2012). To clarify the generalizability of the preceding analyses, we suggest that future quantitative-oriented research examine the overall quality and smoothness of conversational interactions with voice agents that display ASR transcripts compared to agents that do not; for example, by employing the Human–Robot Interaction Conversational User Enjoyment Scale developed by Irfan et al. (2025). Such a comprehensive approach may help determine to what extent ASR transcripts serve as a “lesser evil”—potentially prompting more repair sequences while preventing deeper misunderstandings—or, conversely, whether these transcripts disrupt the usual inner workings of human conversation too severely.

Indeed, as a “feature”, the—systematic—ASR transcripts might belong to a broad category of technologies that endeavor “to remedy a vagueness that is actually required” (Eisenmann et al., 2023), at least, in leisurely conversational interactions. In the two video fragments analyzed above, systematic and non-context-sensitive ASR transcripts hindered intersubjectivity enacted as a “situated, temporarily sustained and only partially shared experience” (Linell and Lindström, 2016). As Linell and Lindström (2016) suggest, experiencing intersubjectivity is dependent on the fact that, in human-human interaction, “a single contribution within a reasonably coherent sequence presupposes an understanding of the prior contribution(s)”. Yet, in the previous fragments, the display of ASR transcripts removed this essential presupposition that one’s previous contribution was understood: when the robot responded verbally, the ASR transcript could override the relevance of the robot’s verbal response *as a response to a specific turn*. In this new configuration, the “weave of interactional moves” (Linell and Lindström, 2016) produced between humans and robots was less likely to offer the (superficial) coherence that is the bedrock of an experience of the ongoing interaction as intersubjectively shared.

## 10 Transcription conventions

### 10.1 Transcription of talk follows Jefferson’s transcription conventions

=         Latching of utterances
(.)       Short pause in speech (<200 ms)
(0.6)     Timed pause to tenths of a second
:         Lengthening of the previous sound
.         Stopping fall in tone
,         Continuing intonation
?         Rising intonation
°uh°      Softer sound than the surrounding talk
.h        Aspiration
h         Out breath
heh       Laughter
((text))  Described phenomena (Jefferson, 2004)

### 10.2 Embodied actions were transcribed using Mondada’s multimodal transcription conventions

** Gestures and descriptions of embodied actions are
      delimited between:
++    two identical symbols (one symbol per participant)
ΔΔ    and are synchronized with corresponding stretches of talk.
*->   The action described continues across subsequent lines
-->*  until the same symbol is reached.
>>    The action described begins before excerpt's beginning.
-->>  The action described continues after the excerpt's end.
…     Action’s preparation.
--    Action’s apex is reached and maintained.
,,,   Action’s retraction.
ric   Participant doing the embodied action is identified in small caps in the margin (Mondada, 2016).

### 10.3 Symbols and abbreviations used in transcriptions refer to the following multimodal dimensions

PA1  Turn at talk from a human participant (PA1, PA2, etc.)
ROB  Turn at talk from the robot
pa1  Multimodal action from a participant (pa1, pa2, etc.)
rob  Multimodal action from the robot
fig  Screenshot of a transcribed event
£    Human’s gaze
*    Human’s arms
@    Human’s whole body
^    Movement in space
$    Robot’s arm
+    Robot’s gaze
%  Robot’s belly screen (what is displayed on its tablet)
#    Position of a screenshot in the turn at talk

## Data Availability

The raw data supporting the conclusions of this article will be made available by the authors, without undue reservation.

## Footnotes

1 These basic observations are independent of whether “understanding” is defined as a mental process or an interactional achievement (Ryle, 1949). Neither the cognitivist nor the anti-cognitivist alternative is directly observable by participants in a conversation.

2 The technical documentation for the Pepper robot’ “speechbar” and the ASR transcript it displays can be found on the website of Aldebaran (formerly Softbank Robotics), the company that designs and manufactures this robot: https://android.aldebaran.com/sdk/doc/pepper-sdk/ch4_api/conversation/conversation_feedbacks.html (retrieved 16 March 2025).

3 The Aldebaran documentation linked above explicitly frames the “speechbar” (including its public transcription of human speech) as a resource intended for humans to get a better grasp of the situation when interacting with Pepper.

4 Partly similar situations include, for example, speaking to someone connected to a polygraph (i.e., a lie detector) or to someone undergoing an MRI scan. In both these situations, a written support makes available processes going on inside someone’s body or brain, which can be used by co-participants as resources to refine their interpretation of this person’s verbal and gestural conduct.

5 The remote ASR service was provided by the Automatic Speech Recognition service of Nuance: https://www.nuance.com/. Upon being sent the continuous flow of sound received by the robot, Nuance returned one or several possibilities (with associated confidence scores) as to what words composed this audio input.

6 For the sake of simplicity, and to obtain this rough estimate of interaction duration, we define the start of the interaction as the participants’ first turn at talk and the end as their final turn at talk. However, identifying the precise onset of a coordinated interaction between the human and the robot would require a more fine-grained analysis (see Holthaus and Wachsmuth, 2021; Rudaz et al., 2023).

7 Some of the gaze points were mapped manually, while others were mapped using Tobii’s automated algorithm. For the automatically mapped points, the “2D mapping layer” (see Figure 4) displays the confidence score: higher values on this graph indicate greater confidence in the algorithm’s accuracy for that specific point. However, all recordings were systematically reviewed by a researcher to ensure the accurate placement of each gaze point on the 2D image. If a gaze point was found to be incorrectly mapped—regardless of the algorithm’s confidence score—it was manually corrected by the researcher. The Tobii I-VT Attention filter was used for this task.

8 The R Studio script used for processing these binned data is based on Richard Andersson’s (Tobii’s Chief Product Owner) script. This script is available at: https://github.com/richardandersson/TobiiProLabScripts/tree/master/plotting%20binned%20metrics.

9 To give one illustrative example of this temporality, Holler and Kendrick (2015) note that in the multi-person interaction settings they studied, “most frequent gaze shifts from current to next speaker were planned around 250 ms and observable around 50 ms prior to turn end.”

10 In other words, for each bin, we computed the average fixation duration on the “ASR Transcript Zone” across all 22 participants, resulting in a single mean value per bin.

11 In this case, for each bin, we subtracted the mean fixation duration on the “Body and Head” zone (averaged across participants) from the mean fixation duration on the “ASR Transcript Zone” (also averaged across participants).

12 Consequently, participant-level differences are not modelled in these particular tests. These results should be interpreted as showing a reliable overall shift of gaze toward the ASR Transcript Zone across the cohort, but further analyses that incorporate random effects for participants would be required to draw conclusions about individual variation.

13 In his well-known criticism of quantification applied to Conversation Analysis’ purposes, Schegloff (1993) suggests that any quantification, if it needs to be done, should be achieved after a careful qualitative analysis of the phenomena studied, so as to avoid aggregating (in the same category) behaviors which were treated as different practices by the members themselves as they were immersed in a local interaction. In the following section, we approach this problem from the other end: we untangle some of the interactional phenomena intertwined in the statistical results.

14 However, since interactions varied in length, etc., this number—while not meaningless—does not lend itself easily to strict or comparable quantification. We rely on the EMCA convention of not emphasizing such figures, as doing so would shift attention away from the phenomenon as “one species” of interactional practice (Ten Have, 2007), regardless of its recurrence.

15 A possible hypothesis is that the ASR transcript may have modified “the limits of what [the parties in a conversation] will seek to bring to determinacy” (Liberman, 1980): that is, what could be “passed” over (Garfinkel, 1967; Liberman, 1980) and left tacit as non-consequential “for current practical purposes” (Albert and Ruiter, 2018; Linell and Lindström, 2016; Schutz, 1972).

16
Ivarsson and Lindwall (2023) remark that “[t]here is no point in imparting the analytic registers of HCI research with conceptual ambiguities by building technical terminology out of vernacular expressions”. Speaking about “understanding” or even “hearing” falls within the scope of this remark on vernacular expressions. Yet, we argue that, here, what the robot “understands” or “hears” is an adequate description of what many participants, emically and locally, were concerned about. Since participants used these terms (when stating that “the robot understood” or “heard” something), we believe they are analytically valid categories emerging from the fragments under study and from our larger corpus.

17 Of course, rather than following a simplistic rule, an optimal solution would be to display an ASR transcript *only when it is interactionally relevant for the co-present members themselves*. However, the local applicability of such a generic guideline for an artificial agent remains uncertain: producing a transcript “only when situationally relevant” confronts the classic problem of relevance and commonsense understanding in AI (McCarthy and Hayes, 1981; Minsky, 1988; Mitchell, 2021).

18 Within a different theoretical framework, a systematically displayed ASR transcript constitutes an exception to a “maxim of minimization” (Levinson, 1987).
